# An Interesting Case of Atherosclerotic Occlusion of the First Septal Perforator in a Physically Young and Fit Individual Causing Complete Heart Block

**DOI:** 10.7759/cureus.3983

**Published:** 2019-01-29

**Authors:** Johnny Chahine, Bicky Thapa, Rama D Gajulapalli, Amer N Kadri, Anjli Maroo

**Affiliations:** 1 Internal Medicine, Cleveland Clinic - Fairview Hospital, Cleveland, USA; 2 Cardiology, Cleveland Clinic - Fairview Hospital, Cleveland, USA

**Keywords:** myocardial infarction, complete heart block, septal perforator occlusion

## Abstract

Complete heart block (CHB) is an unfortunate complication of an anterior and inferior myocardial infarction (MI). We present a case of an atherosclerotic occlusion of the first septal perforator leading to CHB requiring permanent pacemaker placement in a young patient. A 33-year-old healthy white male presented to the emergency department with an episode of syncope. His vitals were stable, and his physical exam was unremarkable. His electrocardiogram (EKG) showed CHB and ST elevations in V1, V2, and V3 suggestive of septal MI. He underwent emergent left heart catheterization which revealed significant stenosis of the proximal left anterior descending (LAD) artery, proximal diagonal artery, and the first septal perforator. An intervention was done with stent placement in the LAD and insertion of a temporary pacemaker. After removal of the temporary pacemaker two days later, the patient developed asystole with alternating bundle branch block which prompted immediate reinsertion of the temporary pacemaker which was replaced later with a permanent pacemaker. The patient was stable afterward and discharged.

The persistent atherosclerotic occlusion of the first septal perforator prevented adequate perfusion of the conduction system, even after revascularization of the proximal LAD. In conclusion, it is essential to acknowledge that difficulty to revascularize an occluded septal perforator raises the need for a permanent pacemaker to prevent a CHB.

## Introduction

Complete heart block (CHB) can be caused by reversible etiologies like myocardial ischemia, electrolyte abnormalities, and medications. CHB is a potential complication of anterior and inferior myocardial infarctions (MI). MI causes high degree atrioventricular block and intraventricular conduction blocks through ischemia or dysregulation of the autonomic system. The incidence of CHB in acute MI varies, ranging between 2.2% and 5% and the incidence of CHB is higher for inferior MI compared to anterior MI [1-2]. CHB is most commonly associated with an occlusion of the right coronary artery (RCA) [3]. The mortality rate of MI is higher if complete heart block is present [1-3]. Thus, prompt recognition of CHB in myocardial infarctions is essential to prevent further deterioration. However, a permanent pacemaker is not indicated for all patients. We present a case of left anterior descending (LAD) artery stenosis along with total occlusion of the first septal perforator, leading to CHB and alternating right bundle branch block (RBBB) and left bundle branch block (LBBB) requiring permanent pacemaker placement in a young patient.

## Case presentation

**Figure 1:**
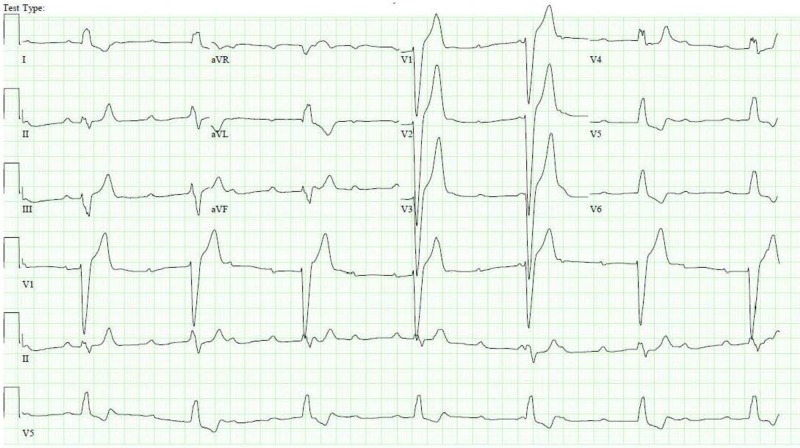
Electrocardiogram showing complete heart block with a wide complex escape rhythm

**Figure 2:**
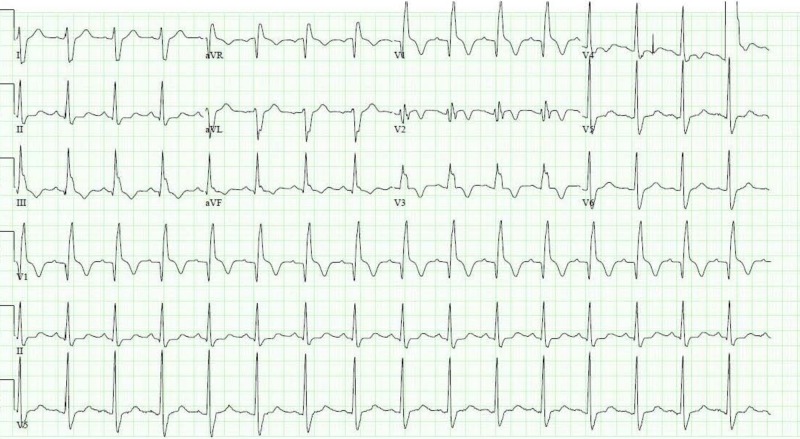
Electrocardiogram showing bifascicular block (complete right bundle branch block and left posterior fascicular block)

A 33-year-old Caucasian male presented to the emergency department with atypical chest pain for the past one week, associated with two episodes of syncope. His past medical history was unremarkable. His family history was relevant for coronary artery disease in his father at the age of 48 years with subsequent coronary artery bypass graft at the age of 52 years. In the emergency department, his electrocardiogram (EKG) showed complete heart block with a wide complex escape rhythm that switched to bifascicular block (RBBB and left posterior fascicular block (LPFB)) (Figures 1-2).

**Figure 3:**
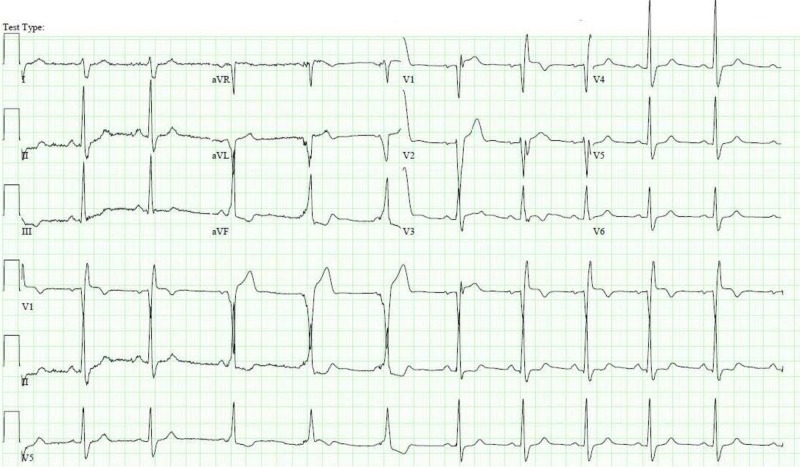
Electrocardiogram showing complete heart block, incomplete right bundle branch block, left posterior fascicular block and ST elevations in leads V1, V2 and V3 suggestive of anteroseptal myocardial infarction

Troponin was 0.72 ng/mL (normal: 0.000-0.029 ng/mL) on admission. A few hours later, his EKG showed ST elevations in leads V1, V2 and V3 suggestive of anteroseptal myocardial infarction (Figure 3).

**Figure 4:**
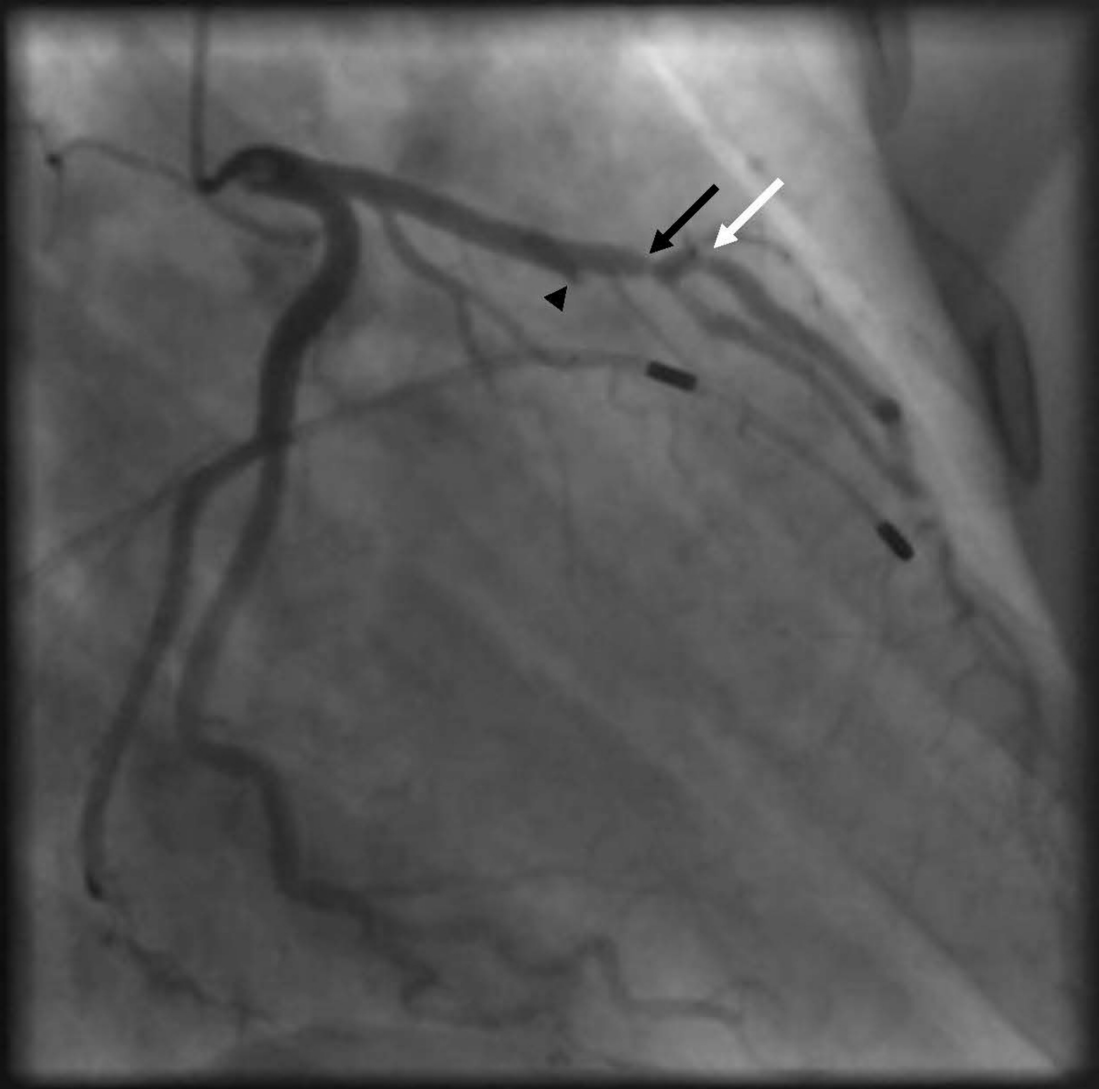
Coronary angiography showing 100% stenosis of the first septal perforator (black arrow head), 90% stenosis of the proximal LAD before the first diagonal (black arrow) and 85% stenosis of the proximal diagonal artery at the level of the bifurcation (white arrow) LAD: left anterior descending

Serial troponins remained elevated. Cardiac catheterization showed 90% stenosis of the proximal LAD before the first diagonal, 85% stenosis of the proximal diagonal artery at the level of the bifurcation, and 100% stenosis of the first septal perforator (Figure 4).

**Figure 5:**
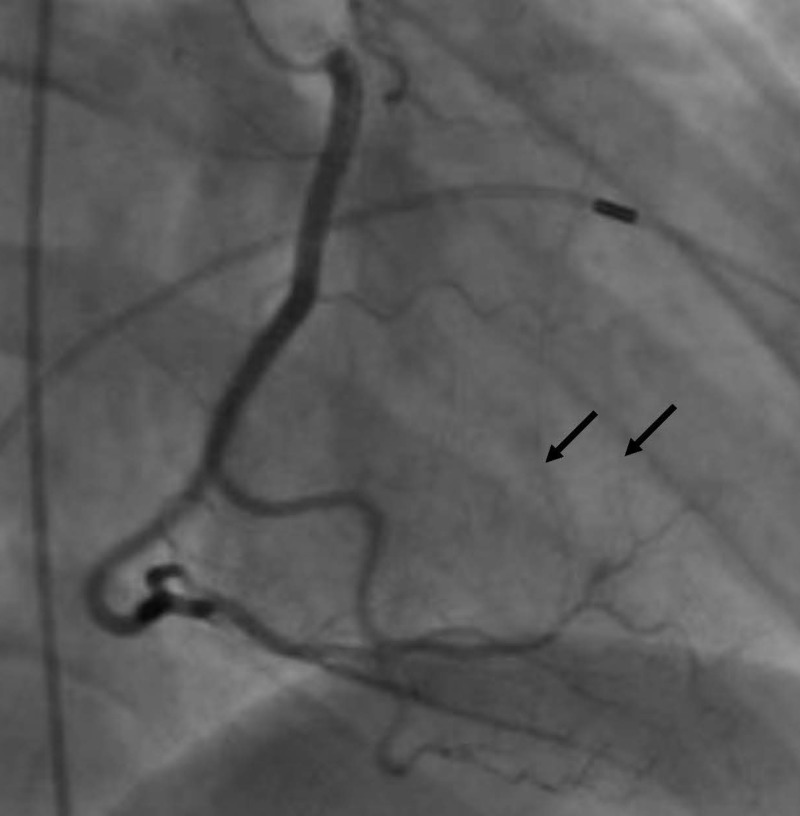
Coronary angiography showing septal collaterals from RCA (black arrows) RCA: right coronary artery

Collaterals from the right coronary artery (RCA) to the first septal perforator were also observed (Figure 5). 

**Figure 6:**
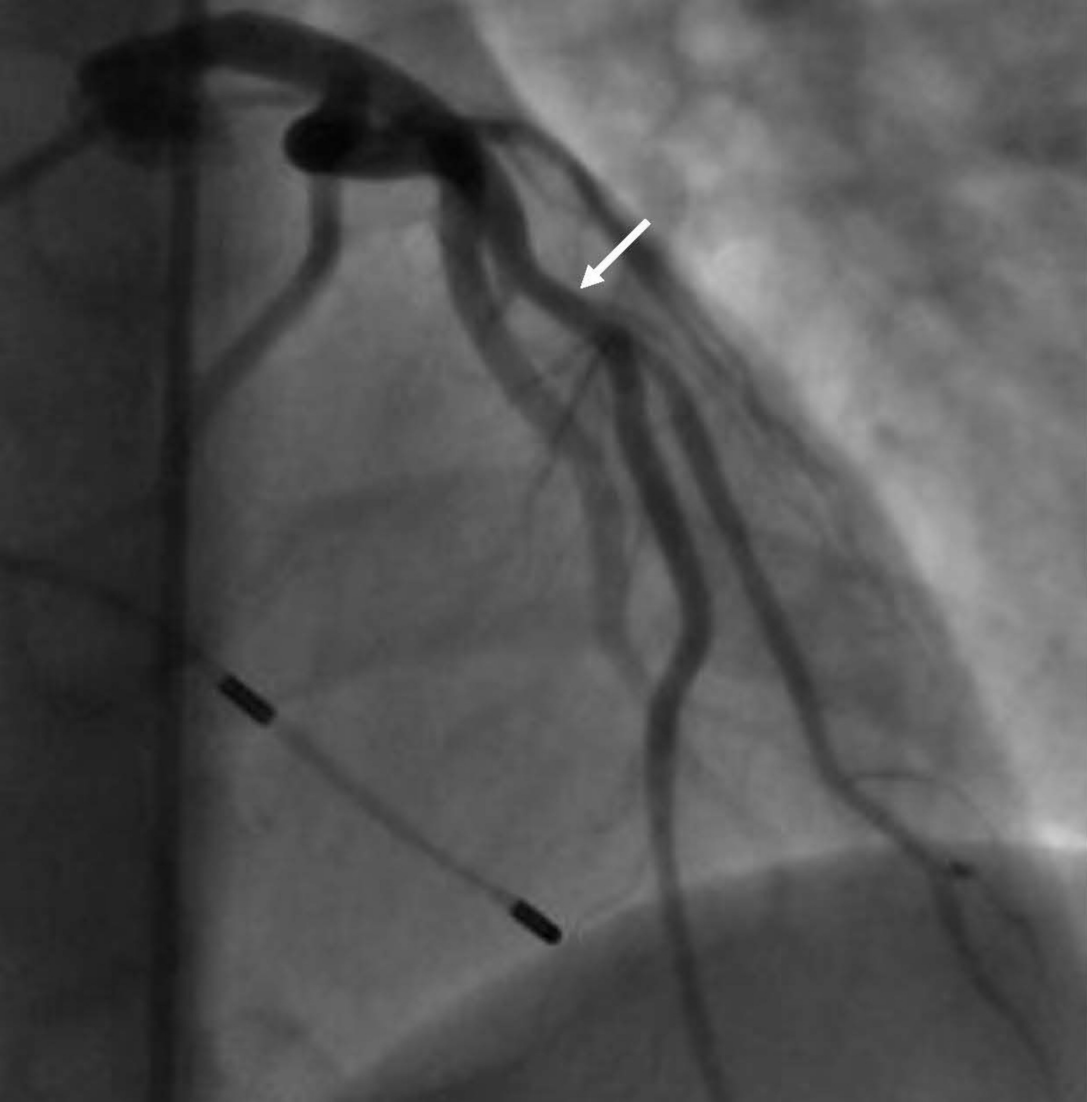
Coronary angiography showing revascularized LAD and diagonal arteries (white arrow) LAD: left anterior descending

A temporary pacemaker wire was inserted. A 2.75 mm x 12 mm Xience drug-eluting stent was placed in the LAD, and balloon angioplasty of diagonal artery was done using a 2.5 mm x 12 mm balloon (Figure 6).

**Figure 7:**
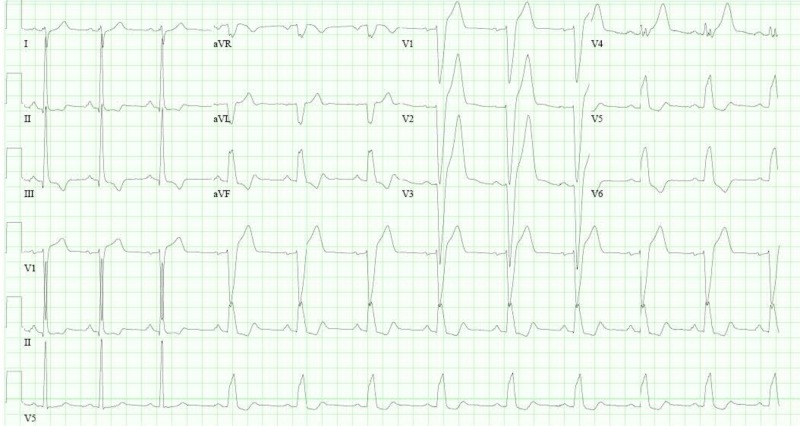
Electrocardiogram showing complete left bundle branch block

**Figure 8:**
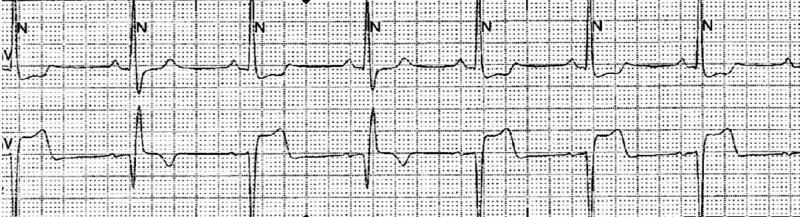
Rhythm strip showing alternating bundle branch block

The patient was treated with aspirin, ticagrelor, metoprolol, lisinopril, and atorvastatin. Echocardiography revealed mild hypokinesis of the basal anteroseptal wall, basal inferior wall, and basal inferoseptal wall with overall preserved ejection fraction. In the subsequent two days, the patient had predominantly one-to-one atrioventricular (AV) conduction with occasional 2:1 AV block. He also had a persistent bifascicular block (RBBB and LPFB). The patient’s temporary pacemaker was removed two days later. However, he developed recurrent episodes of asystole followed by complete LBBB on EKG which required replacement of the temporary pacemaker (Figure 7). The next day, the rhythm strips showed a pattern of alternating bundle branch block (Figure 8).

He underwent insertion of a dual chamber permanent pacemaker, and he was eventually discharged home in stable condition.

## Discussion

Among patients with both myocardial infarction and bundle branch block, the prevalence of RBBB and LPFB is 10% [4]. The septal perforator branches of the LAD supply the anterior two-thirds of the interventricular septum as well as the bundle of His. The posterior third of the septum, the sinoatrial node, and atrioventricular node are supplied, in most cases, by the right coronary artery [5]. Occlusion of the first septal perforator has been reported to cause complete heart block when complicating a percutaneous coronary intervention [6-8]. It is well known that an anterior myocardial infarction complicated with complete heart block is generally associated with an acute reduction in systolic function due to myocardial damage [2]. The “2013 ACCF/AHA (American College of Cardiology Foundation/American Heart Association) guideline for the management of ST-elevation myocardial infarction” recommends temporary pacemaker placement after ST-elevation myocardial infarction for bradycardia with symptoms as well as for “high-grade AV block and/ or new bundle-branch (especially LBBB) or bifascicular block in patients with anterior/lateral MI” (class I, level of evidence C) [9]. As for permanent pacemaker placement, the “2012 ACCF/AHA/Heart Rhythm Society (HRS) focused update of the 2008 guidelines for device-based therapy of cardiac rhythm abnormalities” recommends its installation after acute myocardial infarction for “transient advanced second- or third-degree infranodal AV block and associated bundle-branch block” (class I, level of evidence B) [10].

This case represents an example of the first septal perforator occlusion causing RBBB, LPFB, alternating bundle branch block and complete heart block requiring permanent pacemaker placement. Despite the revascularization of the proximal LAD and the small extent of myocardial damage evident on echocardiography, the patient had a recurrence of his AV block 48 hours after revascularization and removal of the temporary pacemaker. There have been many studies that report the reversibility of high degree atrioventricular block associated with myocardial infarction after revascularization [11-13], even if delayed [14-16]. This was the rationale behind the attempt to remove the temporary pacemaker and potentially avoid an unnecessary permanent pacemaker placement that might limit the patient’s career as a firefighter. In our case, the persistent atherosclerotic occlusion of the first septal perforator prevented adequate perfusion of the conduction system, even after revascularization of the proximal LAD.

## Conclusions

Occlusion of the septal perforators can cause complete heart block after myocardial infarction. Although relatively uncommon, occlusion of a septal perforator may lead to persistent conduction abnormalities that necessitate permanent pacemaker placement. Furthermore, occlusion of a septal perforator may lead to an increase in mortality, even if the LAD is successfully revascularized.
